# The Latest Updates in Swept-Source Optical Coherence Tomography Angiography

**DOI:** 10.3390/diagnostics14010047

**Published:** 2023-12-25

**Authors:** Fan Xia, Rui Hua

**Affiliations:** 1 Department of Ophthalmology, The Fourth People’s Hospital of Shenyang, China Medical University, Shenyang 110000, China; lovemelovetheworld@163.com; 2 Department of Ophthalmology, First Hospital of China Medical University, Shenyang 110001, China

## 1. Introduction

Optical coherence tomography (OCT) is a revolutionary imaging technology in the field of ophthalmic medical imaging. It is a low-radiation, non-invasive, rapid, and high-resolution imaging technique. Since OCT was invented in 1991, it has been widely used for vivo examination of the eye, especially of the fundus [1]. OCT is based on the principle of coherence scanning interferometry, during which reflected and scattered light is collected from tissues at different depths, and the collected scanning duration and the reconstructed signals are analyzed to construct two- or three-dimensional (3D) images. OCT has progressed through three generations (time-domain [TD], spectral-domain [SD], and swept-source [SS] OCT), with improvements in the resolution, penetration depth, speed of scanning, and mode of image acquisition [1]. SD-OCT is by far the predominant imaging modality in ophthalmic clinical practice and is widely accepted by retinal specialists. However, the main limitation of SD-OCT is the imaging depth (1.8–2.2 mm). It is insufficient for ultra-widefield retina imaging, imaging of highly myopic eyes, imaging of posterior scleral staphylomas, and imaging of the anterior segment, all of which are substantially improved with SS-OCT. The latest SS-OCT systems mainly involve vertical-cavity surface-emitting lasers (VCSELs). They surpass SD-OCT in all aspects, including the scanning speed, sensitivity, and imaging depth [2]. They are gradually becoming indispensable in the diagnosis of, and follow-up for, ophthalmic diseases [3].

## 2. Characteristics of SS-OCT

### 2.1. Speed

**Figure 1:**
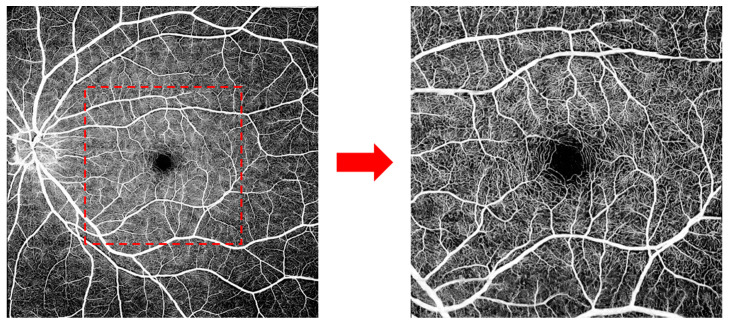
A healthy eye examined via high-resolution, widefield swept-source (SS) optical coherence tomography angiography (OCTA), with a range of 12 × 12 mm. The central area displays copasetic detail of macular capillaries.

The core device in SS-OCT is a frequency-swept laser source, which emits coherent, narrow-band light. It has the advantages of single-point scanning, characteristic of TD-OCT, and fast imaging, characteristic of SD-OCT, which are integral for en face OCT and OCT angiography (OCTA). SS-OCT also has an improved speed of data acquisition and computerized data-processing capabilities. At present, commercial SS-OCT offers speeds of 100,000, 200,000, and 400,000 sweeps per second. In 2019, *Intalight* launched the first device in ophthalmic SS-OCT capable of 200,000 sweeps per second (SVision VG200, SVision Imaging, Ltd., Luoyang, China) to obtain China National Medical Products Administration clearance. *Carl Zeiss* established an ophthalmic SS-OCT device capable of 200,000 sweeps per second (Elite 9000, Carl Zeiss AG, Oberkochen, Germany) in the EU and US five months later. In 2020, *TowardPi* unveiled their ophthalmic SS-OCT system, capable of 400,000 A-scans per second (BM-400K BMizar, TowardPi Medical Technology Ltd., Beijing, China), and started marketing it in China one year later. SS-OCTA provides wider and finer scans than SD-OCTA, and even though its range is several times wider than that of SD-OCTA, local details remain clear when the image is enlarged. Figure 1 is an example of a healthy eye examined via high-resolution, widefield SS-OCTA with a speed of 200,000 sweeps per second and a range of 12 × 12 mm (1024 × 1024 pixels). The central area displays copasetic detail of macular capillaries, which is barely possible with SD-OCTA.

### 2.2. Signal-to-Noise Ratio and Sensitivity

**Figure 2:**
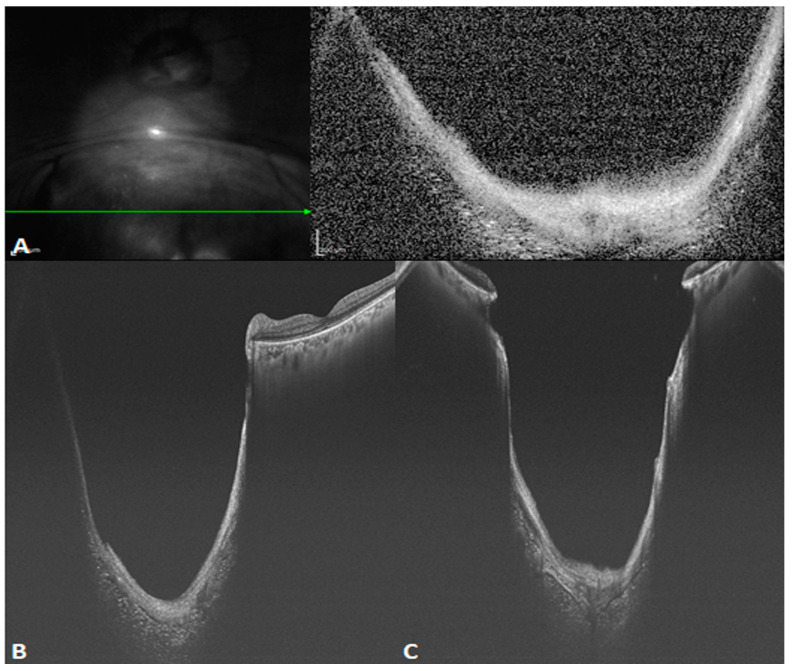
B-scan images of a patient with refractive medium turbidity and a huge choroidal coloboma. (**A**) Spectral-domain OCT (SD-OCT) image showing a part of the coloboma, but the structure is blurry (The green arrow in the left image indicated the orientation of B scan OCT in the right image). (**B**) An oblique SS-OCT scan of the same choroidal coloboma and the macular area. Even the posterior scleral space can be visualized. (**C**) A B-scan SS-OCT image with a range of 20 × 20 mm showing the full extent of the choroidal coloboma.

In SS-OCT, a balanced photodetector is used to reduce the signal loss of light. It has a comparatively facile structure, which efficiently restrains common mode noise. As a consequence, even at the same scanning speed, SS-OCT yields a better sensitivity and higher image signal-to-noise ratio (SNR) than SD-OCT, even in the case of weak OCT signals due to refractive medium turbidity, such as that caused by cataracts or vitreous hemorrhage. This latter shortcoming is overcome by the penetration depth and sensitivity of the technique. Figure 2 reveals the difference between SD- and SS-OCT in acquiring retinal images from a patient with refractive medium turbidity. SS-OCT is better able to display the characteristics of tissue structures, improving clinical diagnosis.

### 2.3. Penetration Depth

**Figure 3:**
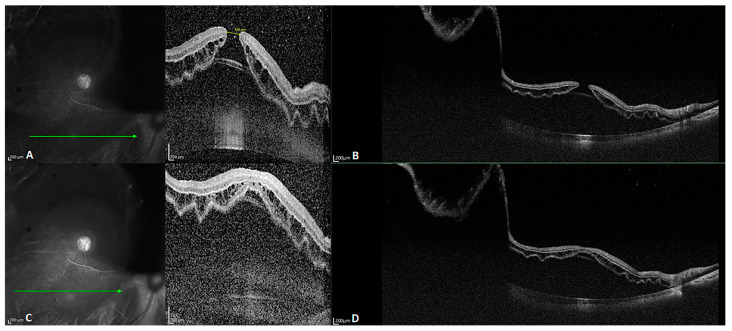
B-scan images of a patient with slightly refractive medium turbidity and a macular hole. (**A**,**C**) SD-OCT images revealing a macular hole and a part of the detached retina; the choroid is not visible because the retina detachment is too high. (**B**,**D**) SS-OCT images with a depth of 6 mm and width of 24 mm, clearly visualizing the retinal detachment, as well as the choroid and part of the sclera.

**Figure 4:**
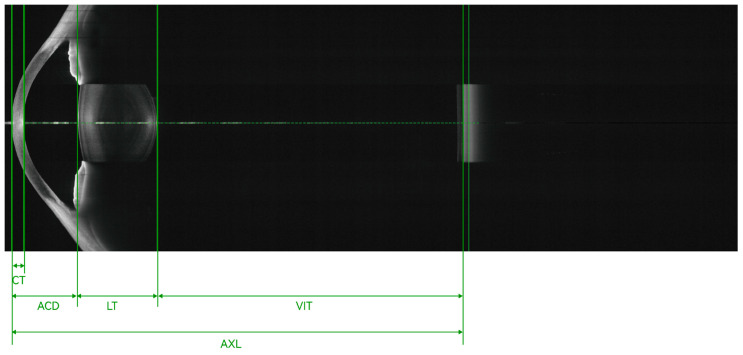
Full-eye, super-depth SS-OCT, achieving an imaging depth up to 40 mm and a 16 mm field of view. CT, cornea thickness; ACD, anterior chamber depth; LT, lens thickness; AXL, axial length; VIT, vitreous.

Biomedical optics requires imaging at a wavelength of 600 to 1000 nm, as tissue absorption is low within this range. SD-OCT is mainly performed at a wavelength of 800 to 900 nm. SS-OCT is mainly performed in the near-infrared radiation spectrum, at wavelengths of 1020 to 1080 nm, as the water absorption coefficient is low at a wavelength of 1060 nm [4], which contributes to better imaging of tissues with a low water content. The improved penetration depth of SS-OCT with less scattering is attributable to its longer wavelength used for imaging. The images in Figure 3 were obtained for a patient with refractive medium turbidity and a macular hole. On the SD-OCT images, the retina and choroidal folds are visible over a certain range, whereas the SS-OCT images display everything from the vitreous to the choroid layer. Currently, most SS-OCT instruments are based on VCSELs, which have a coherence length exceeding 100 mm. Coupled with high-speed data acquisition, they allow steady imaging at a depth of 45 mm in tissue. Therefore, SS-OCT provides a 16 mm field of view in full-eye imaging, as illustrated in Figure 4. This is also the basis for the 3D imaging of the anterior segment.

### 2.4. Field of View

**Figure 5:**
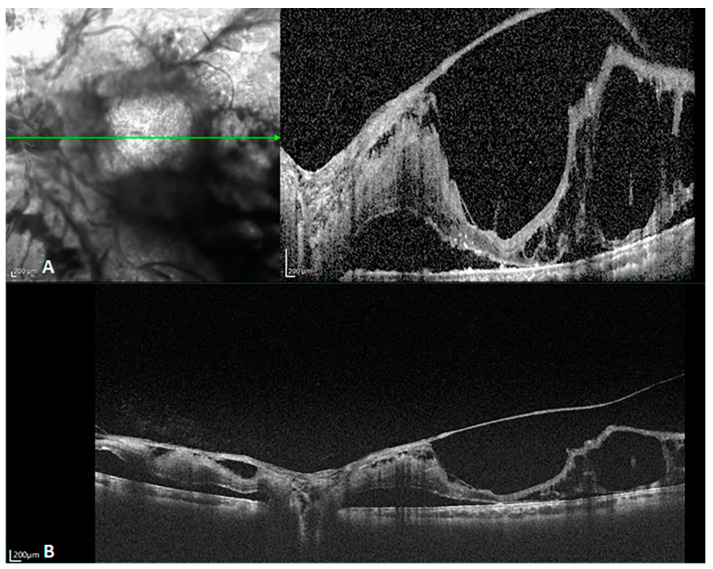
B-scan images of a patient with proliferative diabetic retinopathy. (**A**) SD-OCT showing retinal detachment, with the large proliferative membranes, causing a folding artifact. The choroidal layer is incompletely captured. SD-OCT was unable to capture the whole proliferating membrane and the choroidal tissue below because the retinal bulge was too high (The green arrow in the left image indicated the orientation of B scan OCT in the right image). (**B**) SS-OCT displays an almost identical position. It reveals details of the retina and choroid, even including the choroidal–scleral boundary and part of the sclera. The pre-retinal proliferating membrane extending into the vitreous cavity and the retinal detachment on the nasal side of the optic disk are also clearly visible.

SD-OCT has a limited scope of examinations, and folding artifacts are an inescapable problem because of the technique’s limited imaging depth. When the width of the examined field is expanded, the OCT system requires a deeper range to surround the whole field and avoid folding artifacts. SS-OCT overcomes this problem by enhancing the detection resolution of the interference spectrum. Consequently, it has an impressive range and quality, as demonstrated in Figure 5.

### 2.5. Accurate Segmentation Algorithm

**Figure 6:**
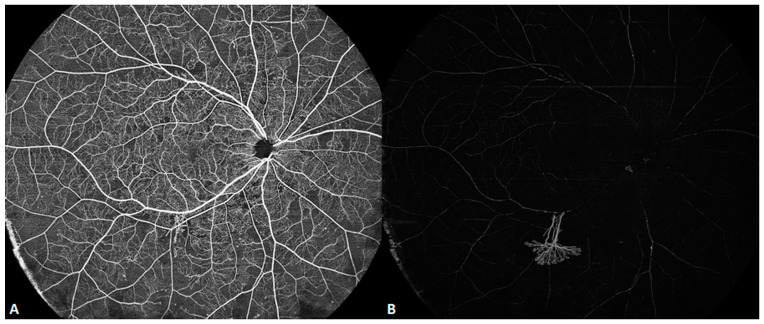
Ultra-wide (24 × 20 mm) SS-OCTA image of a patient with diabetic retinopathy revealing retinal neovascularization at the infratemporal vascular arch, growing into the vitreous. (**A**) Superficial retinal angiographic image. (**B**) Vitreous angiographic image.

**Figure 7:**
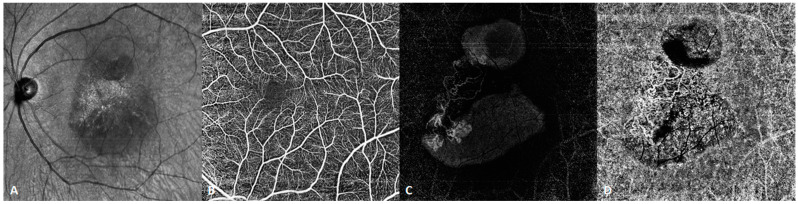
SS-OCTA image of a patient with polypoidal choroidal vasculopathy. (**A**) Infrared fundus image. (**B**) Normal retinal superficial vascular network. (**C**) SS-OCTA demonstrating choroidal neovascularization and a polypoidal lesion under the elevated pigment epithelial detachment (PED) in the avascular layer of the retina. (**D**) The choriocapillaris exhibits a branching neovascular network. SS-OCT clearly reveals the vascular structure within the PED, which was barely revealed using SD-OCT.

OCTA is one of the considerable advances in the OCT field [5]. The main principle of OCTA is the detection of the signal reflection of erythrocyte movement in the vessels. The reflected signal changes as the erythrocytes move, whereas the signal does not change for static tissues. Through scanning the same position at different moments, calculating the differences in the OCT signals, and suppressing the static tissue to the shot-noise limit, the blood vessels are imaged. The segmentation algorithm is indispensable to the overall OCTA algorithm. As the human retina has diverse vascular networks at different layers, each layer needs to be thoroughly examined for an accurate diagnosis. Previously, the segmentation algorithm commonly used was the “graph cut” algorithm. That algorithm is suitable for the segmentation of healthy retinas with continuous and relatively flat layers. However, in pathological cases, layers may be rough, uneven, thinned or thickened, and even fractured. In such cases, the graph cut algorithm may not be able to distinguish between the various layers. SS-OCT is based on a deep learning algorithm and an artificial intelligence (AI) autonomic machine learning model. In terms of computational speed, with the support of high-end graphics processors, it is no less efficient than traditional algorithms. Figure 6 and Figure 7 are two examples of such images. SS-OCTA also provides quantitative metrics, such as the foveal avascular zone, vascular density (VD), and choroidal vascularity index. Accurate quantization is based on accurate segmentation algorithms.

## 3. New Applications of Ultra-Widefield SS-OCT and SS-OCTA

### 3.1. Anterior Segment SS-OCT

**Figure 8:**
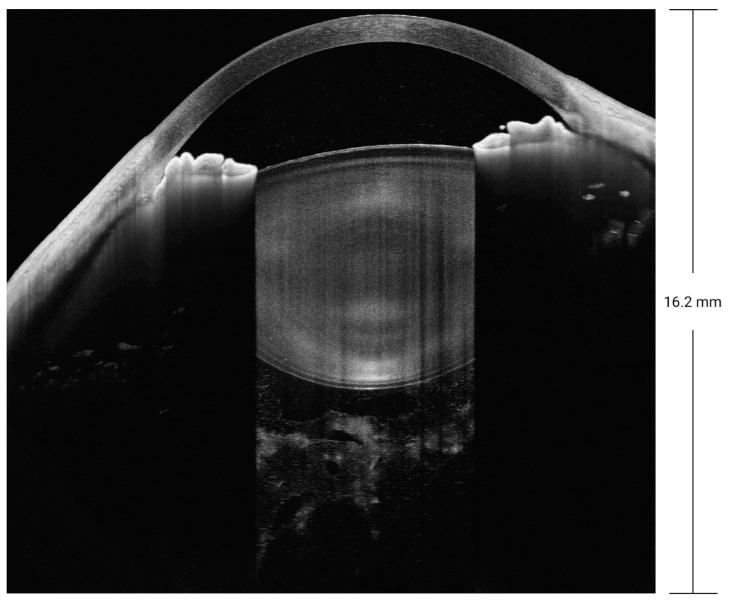
SS-OCT with a depth of 16.2 mm for the anterior segment.

SS-OCT not only enables the examination of the cornea, anterior chamber angle, and iris, as with the previous generations of anterior segment OCT, but also allows the visualization of the entire crystal lens and the vitreous behind it. It yields an unprecedented imaging depth of 16.2 mm for the anterior segment, as demonstrated in Figure 8. SS-OCT examination is faster and more reproducible than previous generations of OCT. For patients with ocular trauma who cannot be examined using ultrasound biomicroscopy, SS-OCT can be used to discover small injuries to the lens and iris that may be overlooked when using slit-lamp examination.

### 3.2. Anterior Segment 3D-OCT

**Figure 9:**
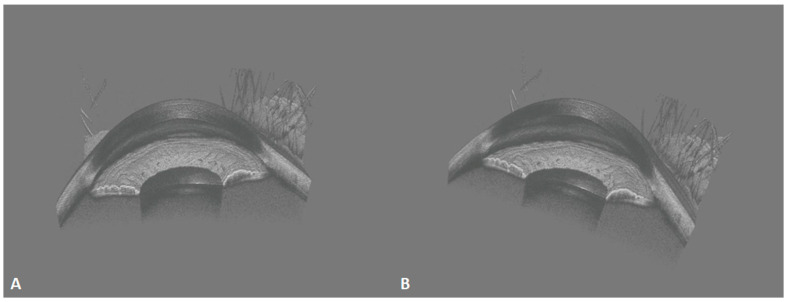
These 400-kHz SS-OCT images demonstrate the normal three-dimensional (3D) anterior segment morphology. (**A**) Frontal view of sagittal plane; (**B**) oblique view of sagittal plane. Iris folds, the anterior chamber angle, and the corneoscleral limbus are clearly visible.

**Figure 10:**
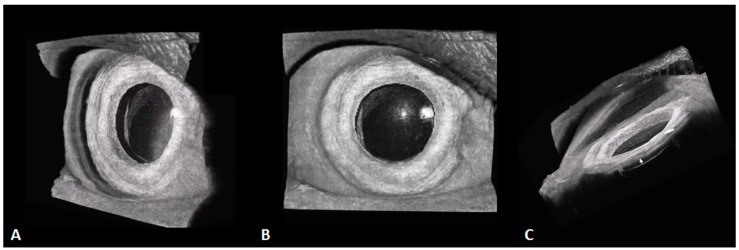
These 3D, 400-kHz SS-OCT images demonstrate an eye with an intraocular lens (IOL) with a small pterygium on the nasal side. (**A**) The structural relationship between the IOL and the iris is clear. (**B**) The frontal view. (**C**) The side view, in which the entire side of the iris is clearly visible (white arrow), as well as the lower interface of the IOL.

The acquisition efficiency of SS-OCT is tremendously improved over that of SD-OCT. That ultrafast speed, together with efficient horizontal and longitudinal scanning, enables the creation of 3D images, as displayed in Figure 9. It allows for a more intuitive and stereoscopic view of the positional relationships between the structures of the anterior segment. The 3D imaging of the anterior segment may greatly improve ophthalmic imaging diagnosis for conditions such as intraocular lens implantation (Figure 10) and anterior chamber space-occupying lesions. Anterior 3D-OCT is helpful in enabling precise customization in refractive surgeries, cataract intraocular lens implantation, and assessing the anterior angle and aqueous outflow for glaucoma diagnosis and monitoring [6].

### 3.3. Anterior-Segment OCTA

**Figure 11:**
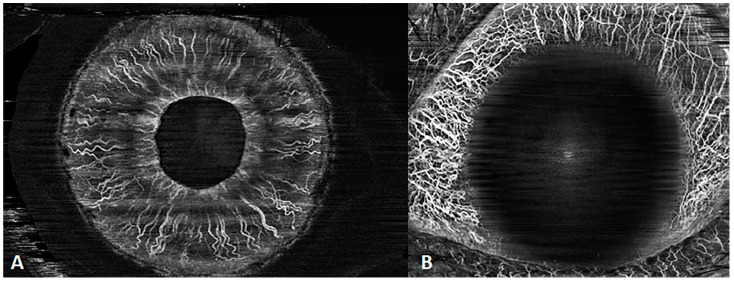
SS-OCTA of the anterior segment. (**A**) Iris angiography; (**B**) conjunctival angiography.

SS-OCTA can penetrate more easily through the dense pigment of the iris than previous generations of OCTA, clearly visualizing the iris flow. In addition, with the help of AI segmentation, this technique easily clarifies the vessels around the limbus and on the iris (Figure 11). Anterior OCTA has a similar ability to indocyanine angiography to reveal corneal vascularization and is superior to fluorescein angiography in revealing iris vascular morphology [7].

**Figure 12:**
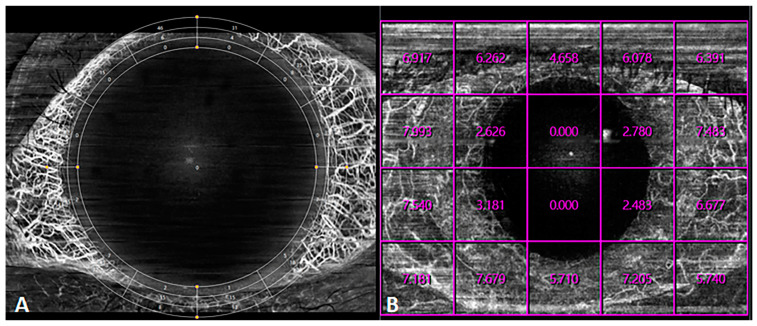
Quantification of anterior-segment OCTA blood flow. (**A**) EDTRS grid mode for the determination of the vascular density. (**B**) Grid mode for the determination of the vascular density. In each lattice, the vascular density is displayed in the corresponding region.

However, quantification via anterior segment angiography is still under exploration. To date, two modalities of quantitative measurements have been developed, “grid” and “EDTRS grid”, each displaying the VD in the grid (Figure 12). However, these modalities do not enable meaningful zoning or provide cues, such as the quantification of the fundus.

### 3.4. Ultra-Widefield OCTA

**Figure 13:**
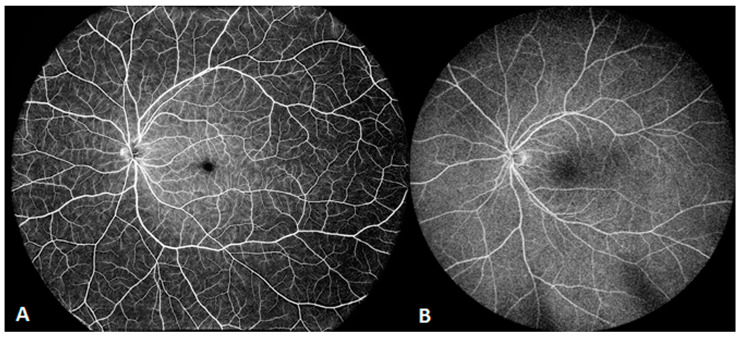
Ultra-wide images of two healthy eyes. (**A**) An ultra-widefield OCTA image of 29 × 24 mm (inner angle = 150°) for a healthy retina with a single scan. (**B**) An ultra-widefield fluorescence angiographic image with an inner angle of 102°.

**Figure 14:**
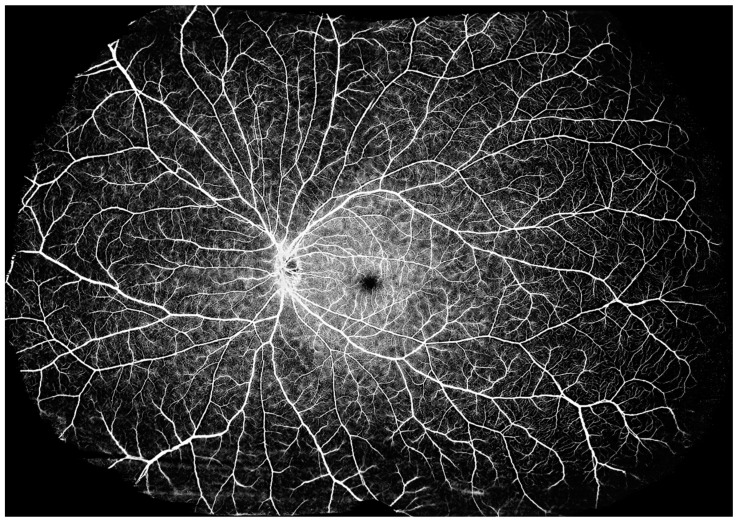
An image of the retina layer as a montage of five ultra-wide angle OCTA images, extending the range to 220° (inner angle). The whole image goes beyond the entire posterior hemisphere, uncovering lesions toward the edge of the retina.

**Figure 15:**
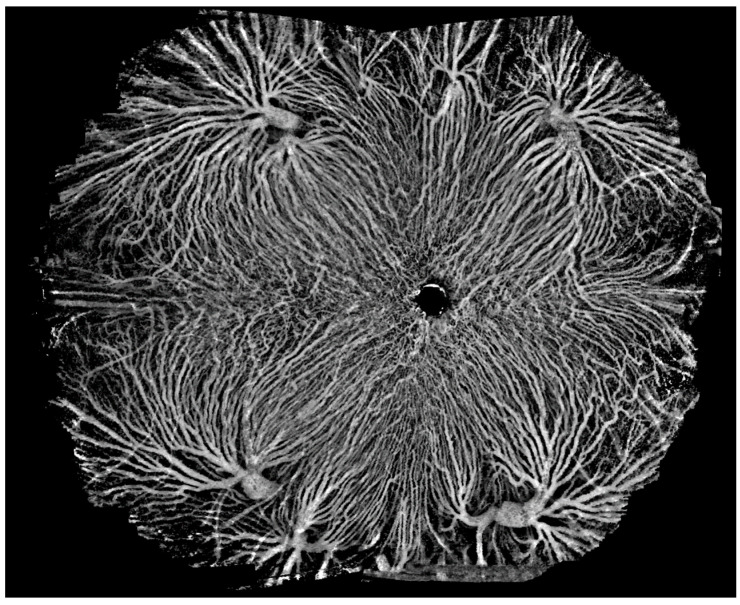
An OCTA image of the choroidal layer as a montage of five ultra-wide angle OCTA images, extending the range to 220° (inner angle). The vortex vein ampullae in all four quadrants are clearly visible.

SS-OCTA modalities with a scanning frequency of 200,000 sweeps per second or more enable ultra-wide-angle imaging. Currently, SS-OCTA modalities with a scanning frequency of 400,000 sweeps per second can yield an image of 29 × 24 mm (inner angle = 150°) with a single scan, which is larger than that yielded using ultra-widefield (102°) fluorescence angiography (Figure 13). A montage of a field of view > 220° may provide much more diagnostic information for diseases characterized by extensive lesions in a non-invasive and more efficient way than imaging with an *Optos-Panoramic* 200 scanning laser ophthalmoscope (Figure 14 and Figure 15). It may also lead to a more complete understanding of the nuanced variations in the human eye.

### 3.5. Optic-Nerve-Head OCTA

**Figure 16:**
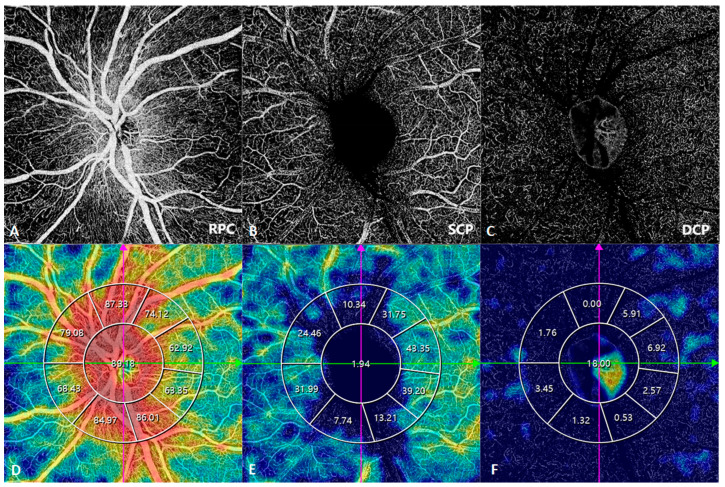
SS-OCTA images of the optic nerve head and corresponding vascular density. (**A**,**D**) Radial peripapillary capillary (RPC). (**B**,**E**) Superficial capillary plexus (SCP). (**C**,**F**) Deep capillary plexus (DCP). Pink and green arrows indicated the orientation of B scan OCT.

Optic-nerve-head OCTA is widely used in the assessment of diseases that cause changes in the optic disc, such as glaucoma, anterior ischemic optic neuropathies, and papilledema. Stratification of the optic disc blood flow via SS-OCTA maintains the traditional OCTA division, including the superficial capillary plexus, deep capillary plexus, and radial peripapillary plexus (Figure 16). It can also be used to quantify the perfusion and VD of circumpapillary regions or the entire optic nerve head. However, the current role of optic-nerve-head OCTA seems to be more supportive and used for follow-up rather than diagnosis [8].

## 4. Future Development

OCT was invented only 30 years ago; as such, it is still a nascent technology compared to traditional imaging modalities, such as ultrasound and computed tomography. It has been applied in clinical practice for less than 20 years, but it has had a revolutionary and long-term impact on ophthalmic imaging diagnosis. The technique is still being improved, with the third generation currently gaining traction. The perfection of the core components will improve the speed, penetration depth, image width, and cost of ophthalmic OCT. Additionally, the rapid progression of AI has also brought revolutionary developments to medical imaging. Many manufacturers have added AI to their image analysis and quantification software as a critical component, optimizing image interpretation. It frees doctors from the routine task of image interpretation and reporting. It can also help human doctors avoid missing precursor lesions or minor lesions.

The two key points that remain to be developed in OCTA are quantification and artifact removal. OCTA blood-flow quantification and related parameters allow doctors to more accurately describe the current status of disease and the level of lesion changes, facilitating meticulous diagnosis, follow-up, and treatment. However, different manufacturers use different algorithms, yielding considerably different results. Moreover, projection artifacts are a critical annoyance that directly affects doctors’ judgment. As the mechanism of artifact formation is very complex, the algorithms currently used to remove them are all based on empirical formulas. A major challenge is the removal of artifacts while retaining key information. With the help of AI, quantitative diagnosis and artifact reduction via angiography are likely to quickly progress.

The retina is the only organ in which the structure of the microcirculation and nerve fibers can be directly observed. OCTA, as the only non-invasive vascular imaging technology, will likely become very valuable in the diagnosis of, and follow-up for, various systemic diseases, especially cardiovascular and cerebrovascular diseases, such as hypertension, hyperlipidemia, and Alzheimer disease [9]. The future prospects for OCT imaging technology are great. Combined with AI, it will bring epoch-making progress in the field of ophthalmic imaging-based diagnosis and will be of great service to humanity.
